# Heavy adolescent drinking makes the adult brain more vulnerable to ethanol by permanently altering the age-dependent interplay between alcohol, GIRK channels and activin

**DOI:** 10.1038/s41380-025-03210-x

**Published:** 2025-09-03

**Authors:** Sophia Stürzenberger, Nicolas Bülow, Liubov S. Kalinichenko, Rebecca Licha, Volker Eulenburg, Marc Dahlmanns, Christian P. Müller, Fang Zheng, Christian Alzheimer

**Affiliations:** 1 Institute of Physiology and Pathophysiology, Friedrich-Alexander-Universität Erlangen-Nürnberg, Erlangen, Germany; 2 Department of Psychiatry and Psychotherapy, Universitätsklinikum Erlangen, Erlangen, Germany; 3 Translational Anesthesiology and Intensive Care, Universität Augsburg, Augsburg, Germany; 4 Institute of Psychopharmacology, Central Institute of Mental Health, Medical Faculty Mannheim, Heidelberg University, Mannheim, Germany

**Keywords:** Neuroscience, Diseases

## Abstract

Adolescent binge drinking is a risk behavior associated with the development of neuropsychiatric disorders later in life, but the pathophysiological mechanisms rendering the adolescent brain vulnerable to the long-term consequences of heavy alcohol consumption are only partially understood. Here, we used a mouse model of adolescent binge drinking and focussed on G protein-gated inwardly rectifying potassium (GIRK) channels which are a molecular target of both ethanol and the pluripotent growth and differentiation factor activin A. In whole-cell recordings from dentate gyrus granule cells in brain slices from alcohol-naive mice, we found a striking reversal of the effect of activin A on ethanol-evoked GIRK current as the mice matured: Whereas activin A reduced the ethanol response in cells from adult mice, the already lower ethanol threshold in cells from young mice was brought down even further by activin A. In cells from adult mice with binge drinking-like experience in their youth, the reversal of the activin effect on ethanol-evoked GIRK current with maturation was abrogated, thereby perpetuating the adolescent phenotype of activin-boosted ethanol sensitivity into adulthood. Underscoring the translational significance of an aberrantly enhanced GIRK current response to ethanol, the GABA_B_ receptor agonist baclofen, which is used as an “off-label” prescription against alcohol use disorders, suppressed the permanently enhanced GIRK response to ethanol after heavy adolescent drinking.

## Introduction

Adolescence is a highly vulnerable period in late brain development that is associated with characteristic behavioral alterations. From a dual systems perspective on risk behavior, incentive processing of rewarding stimuli peaks in mid-adolescence (14–17 years), whereas maturation of cognitive control centers in prefrontal cortex is delayed, continuing well into late adolescence [1]. The staggered maturation of the two systems is thought to account for the enhanced propensity for risk-taking and reward-seeking behavior such as heavy alcohol consumption. Adolescents drink less frequently than adults, but they tend to consume more alcohol on a given occasion [2, 3], conferring distinct risk profiles on adolescent binge drinking and habitual frequent alcohol use [4]. According to SAMHSA´s 2022 National Survey on Drug Abuse, 7.1% and 17.9% of US citizens between 16–17 years and 18–20 years, respectively, exhibit binge use of alcohol, defined as consummating 5+ drinks (females 4 + ) within 2 h on the same occasion on at least 1 day in the last 30 days [2, 5]. The rates of such drinking vary considerably between countries, as reported by the European School Survey on Alcohol and Other Drugs: For individuals aged 15–16 years, binge drinking is three times higher in Germany (28%) and in the United Kingdom (27%) than in France (9%) and the United States (9%) [3]. The Neurobiology of Adolescent Drinking in Adulthood (NADIA) consortium has gathered ample evidence from rodent exposure models that heavy adolescent drinking entails persistent behavioral changes, such as increased anxiety and impulsivity, impaired memory, reduced behavioral flexibility, and altered responses to alcohol, which are associated with changes in functional connectivity and synaptic physiology [6]. Nevertheless, the contribution of individual alcohol targets to the lasting consequences of heavy drinking in the youth remains to be elucidated.

Here, we focused on G protein-coupled inwardly rectifying K^+^ (GIRK) channels (Kir3), an established molecular target of ethanol [7]. GIRK channels in the brain are tetrameric complexes formed by three primary channel subunits GIRK1, GIRK2, and GIRK3, of which only GIRK2 can assemble to functional homomers [8, 9]. Most common in the hippocampus and, with few exceptions, elsewhere in the brain are heteromeric GIRK1/GIRK2 channels [10]. GIRK channels can be constitutively active [11, 12], but they typically serve to translate activation of G_i/o_ proteins by a broad spectrum of neurotransmitters and -modulators into an electrophysiological, i.e. K^+^ current response [8, 9, 13]. Due to the inwardly rectifying current-voltage relationship, GIRK currents have their maximum inhibitory effect on neuronal excitability at membrane potentials around rest and abate with depolarization [10]. Ethanol is capable of opening GIRK channels directly without interacting with G proteins or second messengers [14–18]. Mechanistically, ethanol is thought to stabilize the infrequent spontaneous transitions of the resting channel into the open state by binding to a discrete pocket in the cytoplasmic domain of GIRK2 [19].

Notably, GIRK2-deficient mice displayed increased alcohol consumption and mitigated withdrawal symptoms [20]. In a similar vein, mice with a global knockout of GIRK3 showed an increase in binge drinking and in ethanol reward [21–23]. Because viral expression of GIRK3 in the ventral midbrain restored normal alcohol ingestion in GIRK3-deficient mice, GIRK3 was proposed to gate activation of the mesolimbic dopaminergic pathway [21]. Thus, GIRK3-containing heteromers in the ventral tegmental area (VTA) would be poised to regulate the incentive salience of ethanol. Lending credence to the clinical significance of a GIRK-alcohol partnership, a SNP in the promoter region of the *KCNJ6* gene, which encodes GIRK2, was found to be associated with adult alcohol dependence. Re-assessment of this polymorphism in adolescents with a hazardous drinking record revealed an association that was restricted to those individuals who had experienced psychosocial stress during early life [24]. Finally, in iPSC-derived human glutamatergic neurons from patients with AUD and noncoding SNP variants in the *KCNJ6* gene, intermittent exposure to 20 mM EtOH led to an increased *KCNJ6* mRNA and GIRK2 expression, which normalized neuronal excitability [25]. From a clinical point of view, it is noteworthy that the GABA_B_-receptor agonist baclofen, which opens GIRK channels through a G_i/o_-mediated pathway, is used to treat patients with alcohol use disorders (AUD), although its administration has remained controversial and, except for France, the drug has not gained approval for AUD [26, 27].

Against this backdrop, we first examined the ethanol-evoked GIRK current response in granule cells (GCs) from the dentate gyrus of adolescent and adult mice, and then asked how a period of heavy adolescent drinking followed by a long drug-free interval would affect the neurophysiological profile of GCs and their GIRK current response to ethanol re-exposure in the hippocampus of adult mice. We chose to work on granule cells because, firstly, they are the only neuronal cell type within the hippocampal formation expressing δ subunit-containing GABA_A_Rs. These receptors are present at extra-synaptic sites. They mediate tonic inhibition, and they confer a particularly high sensitivity to low ethanol upon granule cells [28, 29]. Although ethanol´s effect on GABA_A_ receptor-mediated tonic inhibition is not a topic in this study, it seems worth focusing on cells that are already recognized for their high ethanol sensitivity. Furthermore, the present study is complementary to our earlier work on the interaction between ethanol and GABA_A_ receptors that was also performed in the dentate gyrus [30], thereby contributing to a more comprehensive understanding of the multiple effects of ethanol in a particularly sensitive brain region.

In previous work, we reported that the pluripotent growth and differentiation factor activin A, a member of the transforming growth factor β (TGF-β) family, whose multiple effects on synaptic plasticity and GABAergic transmission bears significance for cognition, emotionality and addiction [31–35], also regulates GIRK currents [36] and alcohol response [30]. We therefore used genetic and pharmacological manipulations of activin receptor signaling to interrogate the impact of this factor on the alcohol-GIRK relationship at the two different stages of life. Our findings demonstrate that the effect of activin on ethanol responsiveness normally switches from enhancement to inhibition as the adolescent brain matures. Binge drinking in early ages disables this switch, but the consequences for GIRK channel function in the adult brain can be reversed by baclofen.

## Materials and methods

### Animals

Adolescent (postnatal days, PND, 30–45) and adult (3–5 months old) male wild type (wt) C57BL/6 J mice and transgenic mice expressing a dominant-negative mutant of activin receptor IB (dnActRIB) under the control of the CaMKIIα promoter [37] were used for experiments. Since appreciable activity of this promoter is not observed before the second postnatal week, the transgene is unlikely to interfere with prenatal or early postnatal development. Mice were group-housed (GH) under standard conditions with light/dark cycle (7 am / 7 pm) and free access to water and food. Adolescent alcohol drinking and behavioral tests were performed by experimenters blind to hypothesis and/or genotype, with randomly chosen littermates. All procedures were conducted in accordance with the Animal Protection Law of Germany and the European Communities Council Directive of November 1986 /86/609/EEC), and with approval of local government of Lower Franconia, Bavaria, Germany.

### Adolescent alcohol drinking-in-the-dark (DID; PND 32–45)

Adolescent mice were single-housed (SH) during PND 30–45 and ethanol was made available from PND 32 on during the dark cycle (7 pm to 7 am) using the “two-bottle choice” paradigm, offering one bottle with water and one filled with 20% alcohol. The animals were habituated to two bottles available in each cage for one week before PND 32. Mice were group-housed thereafter with littermates and no further access to ethanol until they were killed in adulthood (3–5 months old) for ex vivo studies. A control group of adolescent mice was single-housed for the same time period but was provided two bottles with water only.

### Blood alcohol concentration (BAC)

Ethanol naive adolescent mice (PND 36–42) were administered an alcohol injection (3.0 g/kg, i.p.). Subsequently, 20 µl blood samples were collected from the submandibular vein at 1, 2, and 3 h after the injection, without the use of anesthesia. These blood samples were immediately mixed with 80 µl of 6.25% (w/v) trichloroacetic acid. After centrifugation, 15 µl of the supernatant were subjected to enzymatic alcohol determination using the alcohol dehydrogenase method, as described elsewhere [30].

### Loss of righting reflex (LORR)

Ethanol naive adolescent mice (PND 36–42) were used for this test. LORR was induced by ethanol injection (3.5 g/kg, i.p.), and observed when the animal became ataxic first and then stopped moving for at least 30 s. The animal was then placed on its back and recovery from ethanol administration was defined as the animal being able to right itself three times within a minute. The latency and duration of LORR were determined.

### Electrophysiological recordings from brain slices

Horizontal brain slices (350 µm thick) containing the dorsal hippocampus were prepared from mice under isoflurane anesthesia, as described previously [36]. Slices were kept in modified artificial cerebrospinal fluid (aCSF) containing (in mM) 125 NaCl, 3 KCl, 1 CaCl_2_, 3 MgCl_2_, 1.25 NaH_2_PO_4_, 25 NaHCO_3_ and 10 d-glucose at room temperature for at least 2 h before recording. Individual slices were then transferred to a submerged recording chamber perfused with standard aCSF (divalent cations now set to 1.5 mM MgCl_2_ and 2.5 mM CaCl_2_) at 31 ± 1 °C. All solutions were constantly gassed with 95% O_2_ - 5% CO_2_.

Whole-cell recordings were performed from visually identified DG granule cells (GCs) of the suprapyramidal blade located in the outer part of the granule cell layer (i.e. close to the molecular layer). Patch pipets were filled with (in mM) 135 K-gluconate, 5 HEPES, 3 MgCl_2_, 5 EGTA, 2 Na_2_ATP, 0.3 Na_3_GTP, 4 NaCl (pH 7.3). To avoid sampling immature granule cells, only GCs with membrane input resistance < 400 MΩ were included. In voltage-clamp mode, series resistance was about 6–15 MΩ and compensated by 60–80%. In tetrodotoxin (TTX, 0.5–1 µM), voltage ramps from −50 to −140 mV at a rate of 0.1 mV/ms were used to determine current-voltage (I-V) relationships. To investigate the effect of activin on GIRK channel activity, slices were incubated with recombinant activin A (25 ng/ml; R&D System, Minneapolis, MN, USA). Preincubation with recombinant activin A started 30 min after slice preparation and lasted for 3–6 h, a protocol which we had adapted previously [36, 38] to mimic the transient surge in endogenous activin A in mice briefly exposed to an enriched environment. Slices not preincubated with recombinant activin A were maintained in a storage chamber for up to 8 h before recording to exclude a systematic bias towards shorter storage times in the control group. In current-clamp mode, action potentials (AP) were elicited by a depolarizing ramp protocol (from 0 to 100 pA in 2 s) starting from −70 mV preset by DC injection. All potentials were corrected for liquid junction potential (10 mV). Signals were filtered at 6 kHz (for current clamp) or 2 kHz (for voltage clamp) and sampled at 20 kHz using a Multiclamp 700B amplifier in conjunction with Digidata 1440 A interface and pClamp10.6 software (Molecular Devices, Sunnyvale, CA). MiniDigi 1 A and AxoScope 10.6 were used for low-resolution scope recording, sampled at 1 kHz (Molecular Devices, Sunnyvale, CA). To ensure adequate power to detect the effect of treatment, individual parameters were sampled from at least 4 animals in each group, with sample size of 6–12 cells.

Unless otherwise stated, drugs and chemicals were obtained from Tocris Bioscience (Bio-techne GmbH, Wiesbaden, Germany) and Sigma-Aldrich Chemie GmbH (Steinheim, Germany). Alcohol stock solution (5 M) was prepared just before recording and kept on ice.

### Enzyme-linked immunosorbent assay (ELISA)

Adolescent mice, and adult mice with and without adolescent treatment, were sacrificed under anesthesia with isoflurane and the brain was quickly dissected out. The isolated hippocampus was homogenized in lysis buffer containing 0.32 M sucrose, 5 mM Tris-HCl (pH 8.0) and a protease inhibitor cocktail. Homogenates were centrifuged at 13,000 x g at 4 °C for 10 min (twice). Supernatant was collected for assaying levels of activin A and ActRIB according to the manufacturer’s instructions, with ELISA kits from R&D Systems (Activin A Quantikine ELISA Kit; Minneapolis, MN, USA) and ELAab Science Co. Ltd (Activin Receptor Type IB ELISA Kit; Wuhan, China), respectively.

### Reverse transcription quantitative real-time PCR (RT-qPCR)

Dentate gyrus from 4 dorsal hippocampal slices was trimmed out, collected and stored at −80 °C. RNA was then isolated according to the manufacturer’s protocol (RNeasy Plus Universal Mini Kit; QIAGEN, Hilden, Germany), reverse-transcribed into cDNA (High-Capacity cDNA Reverse Transcription Kit; Applied Biosystems, Waltham, MA, USA), and stored at −20 °C. RNA levels were determined using RT-qPCR according to the manufacturer’s instructions (ABsolute QPCR Mix SYBR Green no ROX, Thermo Fisher Scientific, Waltham, MA, USA) in a realple × 4 cycler (Eppendorf, Hamburg, Germany). The following primers were used (Eurofins Genomics, Ebersberg, Germany): (1) GIRK1, 5′-ctctcggacctcttcaccac-3′ (forward) and 5′-gccacggtgtaggtgagaat-3′ (reverse), (2) GIRK2a, 5′-acctgacggacatcttcacc-3′ (forward) and 5′-aatcagccaccagatcatcc-3′ (reverse), and (3) TATA binding protein (TBP; 146 bp), 5′-gccaagagtgaagaacaatcc-3′ (forward) and 5′-ccttccagccttatagggaac-3′ (reverse). Every biological sample was measured as a technical duplicate and then averaged. Then, ΔCq values were calculated by subtracting the GIRK1 and GIRK2 from the TBP values. ΔΔCq values for both Girk1 and Girk2 mRNAs in wt and dnActRIB were calculated separately by normalizing the respective ΔCq values to their respective Girk1 and Girk2 values. Finally, 2^−ΔΔCq^ values were calculated and displayed.

### Statistical analysis

Electrophysiological data analysis was performed off-line with Clampfit 10.6 (Molecular Devices, CA, USA). Data are expressed as means ± SEM. OriginPro 2018G (OriginLab Corporation, Northampton, MA, USA) was used for statistics and graphs. Shapiro-Wilk test was used to assess normality of data distribution, and the null hypothesis was accepted when *p*-value was larger than 0.05. Statistical comparisons were performed using unpaired or paired Student’s t-test and one-way or two-way analysis of variance (ANOVA) followed by Tukey’s post-hoc test, as appropriate. Significance was assumed for *p* < 0.05.

## Results

### Effect of activin A on ethanol-induced K^+^ current reverses as adolescent granule cells enter adulthood

**Fig. 1:**
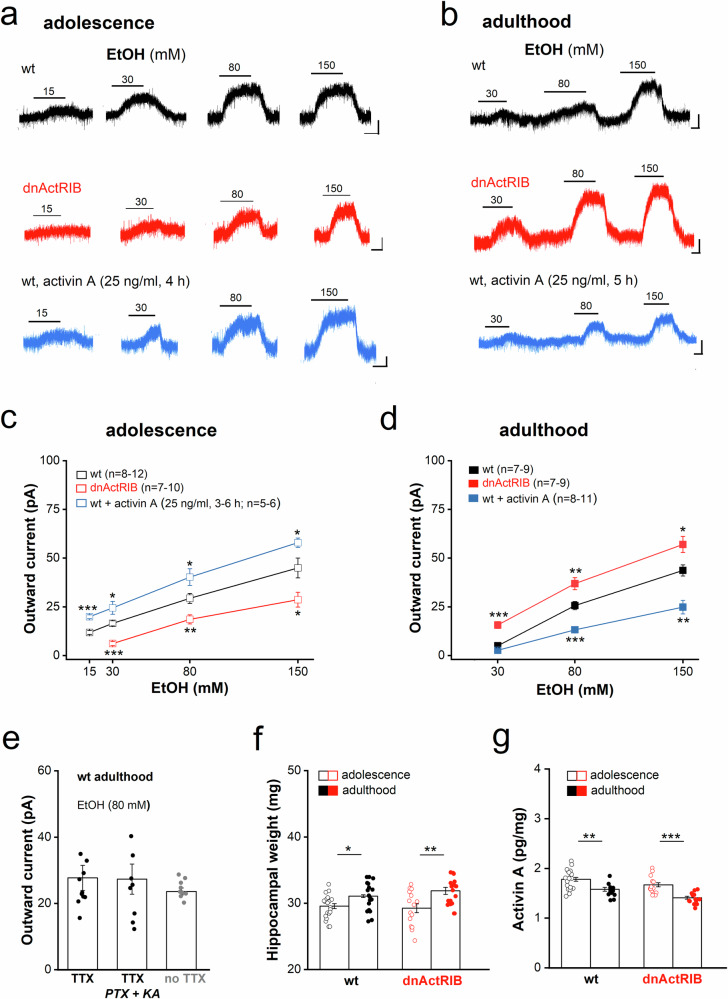
Effect of activin A on EtOH-induced outward current in DG GCs reverses with transition from adolescence to adulthood. **(a-b)** In whole-cell voltage-clamp recordings (V_h_ −70 mV) from GCs in brain slices of adolescent **(a**) and adult mice (**b**), increasing EtOH concentrations induced outward currents in a reversible, dose-dependent fashion. Recordings were made in slices from wt mice (black traces) and from dnActRIB mice (red traces), and in wt slices incubated with recombinant activin A (3–6 h, 25 ng/ml, blue traces). Scale bars: 2 min, 20 pA. (**c-d**) Dose-responses curves show outward current amplitude as function of EtOH dose for the recording conditions illustrated above. (**e**) Comparison of EtOH-induced outward current in GCs from adult wt mice recorded in TTX alone (left column) or in combination with blockers of ionotropic GABA and glutamate receptors (picrotoxin, PTX, 100 µM; kynurenic acid, KA, 2 mM, middle column), or without any blocker (right column). (**f-g)** Columns show hippocampal weight in adolescence and adulthood (**f**), and the normalized levels of activin A protein in hippocampus as determined by ELISA in the two age groups (**g**). Statistical comparisons were performed using one- or two-way ANOVA followed by Tukey’s post-hoc test (**c-e**) or two-tailed student’s t-test (**f-g**). * *p* < 0.05; ** *p* < 0.01; *** *p* < 0.001.

In whole-cell voltage-clamp recordings from mature granule cells (GCs) in dorsal hippocampal slices from adolescent wt mice (PND 36–42), escalating concentrations of ethanol (EtOH, 15, 30, 80, 150 mM, added to the bath solution) gave rise to reversible outward currents, with a threshold below 15 mM (Fig. 1a, c). The concentrations of ethanol used in our study should reflect a realistic scenario as ethanol was found to affect brain function across a range from low millimolar to 100 mM in naïve and occasional users [7]. Outward currents were accompanied by a significant reduction in membrane input resistance (R_N_). In 80 mM EtOH, R_N_ declined from 245.4 ± 21.2 MΩ to 185.0 ± 16.1 MΩ (*n* = 12; *p* = 5.29e^−4^, paired t-test).

In GCs from adolescent transgenic mice expressing a dominant negative mutant of activin receptor IB (dnActRIB), which begins to disrupt activin signaling only after the mice have reached the second postnatal week so that the transgene is unlikely to interfere with prenatal or early postnatal development [37], EtOH sensitivity was significantly dampened, causing a downward shift of the dose-response (D-R) curve (Fig. 1a, c). Vice versa, incubation of adolescent wt slices with recombinant activin A (25 ng/ml) for 3–6 h shifted the D-R curve upwards (Fig. 1a, c). We chose to administer activin A for 3–6 h, as this time window was the best compromise, on the one hand mimicking the rather brief surge in endogenous activin A in response to internal or external stimuli delivered in vivo [31, 38, 39], and on the other hand taking into account the temporal constraints of keeping brain slices at the same level of viability over time. Surprisingly, the relationship between activin signaling and EtOH sensitivity was fully reversed as mice reached adulthood: Now, GCs from adult dnActRIB mice displayed significantly enhanced EtOH responses, whereas incubation with activin A caused a marked decline in current responses (Fig. 1b, d). The EtOH current was an intrinsic property of the cells, as it remained unchanged when the cells were pharmacologically isolated from synaptic inputs (Fig. 1e).

The striking developmental reversal of the effect of activin A on EtOH currents was accompanied by a change in the level of endogenous activin A protein with maturation of the hippocampus. Normalized to the respective weight of the hippocampus (Fig. 1f), the level of activin A in the hippocampus of both mouse lines exhibited a small, but significant decrease with maturation (Fig. 1g), whereas the expression of its major signal-transducing receptor subtype IB (ActRIB) was comparable between young and mature hippocampus (adolescent-wt 85.7 ± 2.7 pg/mg, *n* = 14; adult-wt 78.4 ± 2.3 pg/mg, *n* = 8; *p* = 0.082).

### Alcohol targets G protein-gated inwardly rectifying potassium (GIRK) channels in an activin-dependent fashion

**Fig. 2:**
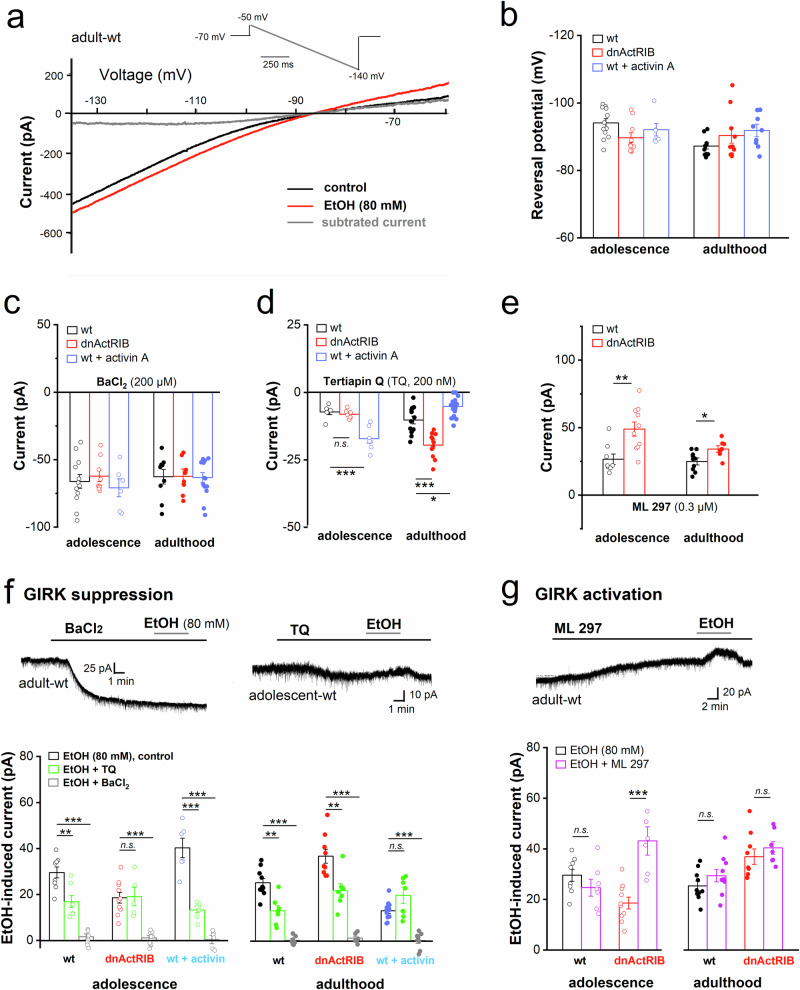
Effect of activin A on standing and EtOH-evoked inwardly rectifying K^+^ current in GCs from adolescent and adult mice. (**a**) Effect of EtOH (80 mM) on current-voltage (I-V) relationship in adult wt cell was determined using a voltage ramp from −50 mV to −140 mV (inset above). EtOH-induced current (grey trace) was calculated by subtracting I-V curve before (black trace) from that during maximum effect of EtOH (red trace). (**b**) Reversal potentials of EtOH-induced currents were determined from I-V curves as shown in (**a**) and summarized in the histogram for the different experimental groups indicated in like-colored letters above columns. (**c-e**) Shifts in holding current (Vh −70 mV) in response to BaCl_2_ (200 µM; **c**), tertiapin Q (TQ, 200 nM; **d**), and ML 297 (0.3 µM; **e**). **(f-g)** Summary of acute alcohol-induced current in the presence of the two current blockers (**f**) and the current activator (**g**). Current traces above depict the respective effects of BaCl_2_, TQ and ML 297 on holding current and alcohol response. Statistical comparisons were performed using one-way ANOVA followed by Tukey’s post-hoc test (**b, c, d, f**) **or** an unpaired, two-tailed student’s t-test at α = 0.05 (**e, g**). *n.s*. not significant; * *p* < 0.05; ** *p* < 0.01; *** *p* < 0.001.

In GCs of young and mature wt and transgenic mice, voltage ramp-evoked currents measured in the absence and presence of EtOH intersected and reversed near K^+^ equilibrium potential, indicating that they were carried by K^+^ channels (Fig. 2a, b). Across all preparations, bath application of low BaCl_2_ (200 µM), which exerts a fairly selective block of inwardly rectifying K^+^ (IRK) channels [40], gave rise to an apparent inward current of roughly equal amplitude, most likely resulting from suppression of a standing (tonic) IRK current (Fig. 2c). With IRK conductance blocked by low Ba^2+^, EtOH (80 mM) failed to evoke the characteristic outward current (Fig. 2f).

To directly examine the interaction of EtOH with GIRK channel, we employed tertiapin Q (TQ) and ML 297 (VU0456810), which specifically block and activate, respectively, GIRK1-containing channels [41, 42]. As with low BaCl_2_, TQ had effects on its own (Fig. 2d). Application of TQ (200 nM) induced a small inward current in wt GCs, which did not differ significantly between the two age groups (adolescent-wt TQ: −7.2 ± 1.0 pA, *n* = 6; adult-wt TQ: −10.3 ± 1.4 pA, *n* = 12, *p* = 0.190; Fig. 2d). The apparent inward current in TQ most likely results from suppression of a standing (tonic) GIRK current that is maintained by ambient adenosine and other neuromodulators [43, 44]. As an interesting additional finding, our recordings showed that in GCs of dnActRIB mice, in which activin receptor signaling is disrupted from the second postnatal week on [37], tonic GIRK current is only enhanced in neurons of adult mice (adult-dnActRIB TQ: −19.5 ± 1.4 pA, *n* = 11; *p* = 1.68e^−4^ vs adult-wt TQ), but not in those of adolescent ones (adolescent-dnActRIB TQ: −8.1 ± 0.6 pA, *n* = 7; *p* = 0.493 vs adolescent-wt TQ) (Fig. 2d). Application of recombinant activin A (25 ng/ml for 3–6 h), in turn, strongly increased TQ-sensitive current in GCs of young wt mice (adolescent-wt TQ in activin, −17.2 ± 1.6 pA, *n* = 7; *p* = 7.27e^−4^ vs adolescent-wt TQ), whereas in GCs of adult wt mice, TQ-sensitive current was smaller compared to wt controls (adult-wt TQ in activin, −5.5 ± 0.9 pA, *n* = 17; *p* = 0.006 vs adult-wt TQ) (Fig. 2d, blue columns). These findings suggest that the effect of activin on GIRK current depends on the life stage, with a rise in activin level promoting GIRK current in adolescence but causing a reduction in adulthood. GCs in young and adult wt slices displayed almost identical pools of readily activatable GIRK channels upon ML 297 (0.3 µM) application, as indicated by a comparison of the drug-induced outward currents (adolescent-wt, 26.6 ± 3.8 pA, *n* = 8; adult-wt, 24.9 ± 2.6 pA, *n* = 10, *p* = 0.696; Fig. 2e, black columns). Compared to their wt counterparts, dnActRIB GCs from both age groups exhibited stronger outward currents in response to the same low concentration (0.3 µM) of ML 297 (adolescent-dnActRIB, 48.9 ± 5.2 pA, *n* = 10, *p* = 0.005 vs adolescent-wt; dnActRIB-adult 34.1 ± 2.4 pA, *n* = 7, *p* = 0.016 vs adult-wt, *p* = 0.040 vs dnActRIB-adolescent) (Fig. 2e, red columns). Notably, the small, but significant difference in ML 297-activatable GIRK channels between wt and mutant GCs during adulthood disappeared, when the compound was applied at higher concentrations (1 and 10 µM; Supplementary Fig. 1a). In GCs from adolescent transgenic mice, however, ML 297 induced significantly stronger currents at all concentrations, suggesting that the loss of activin signaling leads to a higher density of ML 297-sensitive GIRK channels in GCs from young mice compared to those from older ones (Supplementary Fig. 1a).

We next explored the effect of TQ and ML 297 on the outward current induced by 80 mM EtOH (Fig. 2f, g). In adult slices of either genotype, TQ strongly reduced the EtOH current (Fig. 2f, right). When adult wt slices were preincubated with activin (25 ng/ml for 3–6 h), which suppressed TQ-sensitive current (Fig. 2d, right), TQ failed to affect EtOH response (Fig. 2f, right). In wt GCs from adolescent mice, TQ produced a similar reduction of EtOH current (adolescent-wt, EtOH in TQ, 16.9 ± 2.6 pA, *n* = 7, *p* = 0.005 vs adolescent-wt EtOH alone), which was more pronounced following activin incubation (25 ng/ml for 3–6 h, Fig. 2f, left; adolescent-wt, EtOH in TQ and activin, 12.4 ± 1.1 pA, *n* = 8, *p* = 1.51e^−5^ vs adolescent-wt EtOH in activin). In contrast, TQ failed to alter the already smaller EtOH response in mutant GCs (adolescent-dnActRIB, EtOH in TQ and activin, 17.7 ± 3.5 pA, *n* = 6, *p* = 0.835 vs adolescent-dnActRIB EtOH alone; Fig. 2f, left). Interestingly, ML 297 (0.3 µM) did not affect the EtOH response (80 mM) in GCs of either adult genotype (Fig. 2g; adult-wt EtOH in ML 297: 29.5 ± 2.4 pA, *n* = 10, *p* = 0.268 vs adult-wt EtOH alone; adult-dnActRIB EtOH in ML 297: 40.3 ± 2.5 pA, *n* = 7, *p* = 0.414 vs adult-dnActRIB EtOH alone), nor in GCs of adolescent wt mice (24.9 ± 3.3 pA, *n* = 7, *p* = 0.307 vs adolescent-wt EtOH alone). However, when examined in GCs of young transgenic mice, ML 297 pretreatment produced a striking surge in EtOH sensitivity, amounting now to 41.4 ± 6.5 pA (*n* = 6, *p* = 1.83e^−4^; Fig. 2g). Thus, preceding recruitment of GIRK channels in GCs from young mutant mice elevated the subsequent EtOH current to the level observed in GCs of their adult counterparts (*p* = 0.398). In other words, ML 297 confers an adult-like alcohol response pattern upon neurons of adolescent mice.

### Adolescent binge drinking enhances GIRK current response to acute alcohol re-exposure later in life

**Fig. 3:**
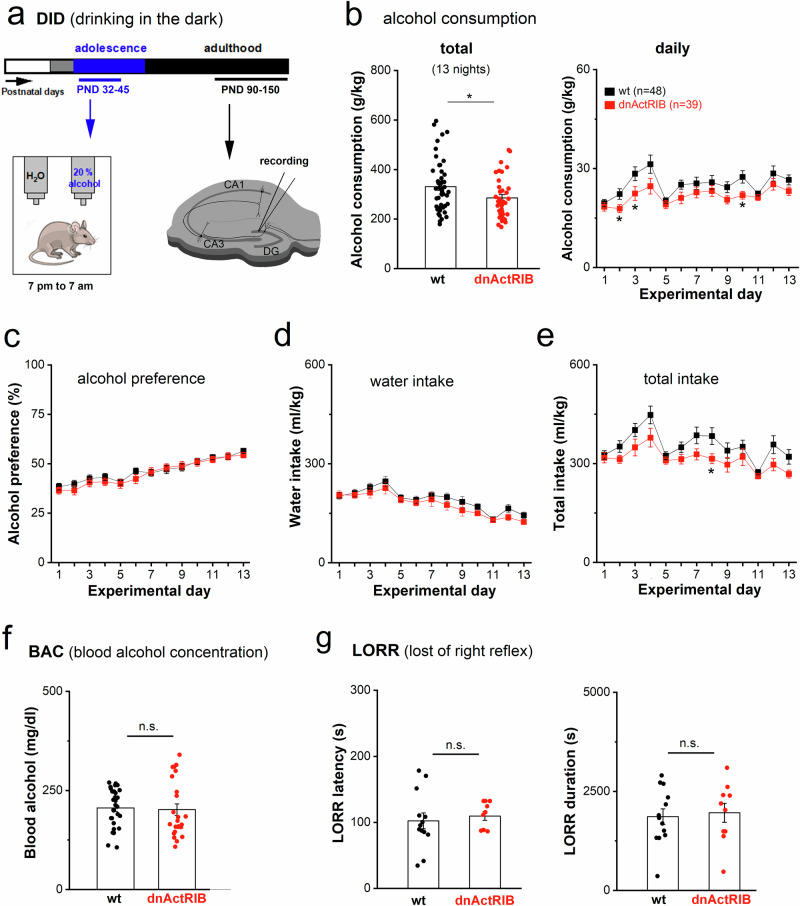
Adolescent binge drinking model (drinking-in-the-dark, DID) reveals lower ethanol consumption in dnActRB mice compared to wt mice. (**a**) Timeline of experiments using DID model. Mice were single-housed (SH) during postnatal day (PND) 30–45, with or without access to 20% alcohol (from PND 32 on) during their active dark cycle (7 pm to 7 am). (**b**) Total (left) and daily consumption (right) of alcohol (20%) over 13 nights in wt and dnActRIB mice. (**c-e**) Quantification of relative preference for alcohol over water (**c**), water intake (**d**) and total intake (**e**). (**f**) Blood alcohol concentration (BAC), determined within 3 h after alcohol injection (3.0 g/kg, i.p.) und expressed as area under the curve (AUC), was comparable in wt and dnActRIB adolescents. (**g**) Loss of righting reflex (LORR) latency to sedation and LORR duration after an acute alcohol application (3.5 g/kg, i.p.). Statistical comparisons were performed using an unpaired, two-tailed student’s t-test at α = 0.05 (**b,** **f,** **g**) or two-way ANOVA followed by Tukey’s post-hoc test (**b-e**). * *p* < 0.05.

To unveil persistent neurophysiological effects after heavy adolescent drinking, we adapted the drinking-in-the-dark paradigm (DID) [45] with the timeline depicted in Fig. 3a. When summed over the entire DID period, dnActRIB mice consumed less alcohol (dnActRIB-adolescents, 285.5 ± 13.9 g/kg, *n* = 39) than their wt littermates (wt-adolescents, 330.4 ± 15.4 g/kg, *n* = 48, *p* = 0.037; Fig. 3b). Plots of daily alcohol consumption revealed an initial peak that was delayed and smaller in transgenic mice compared to wt mice (Fig. 3b). Throughout the entire DID period, the relative alcohol preference did not differ between the two mouse lines (Fig. 3c–e). After DID, mice were group-housed again with their littermates, and the growth of mice was monitored weekly until adulthood (PND 90; Supplementary Fig. 2). Measurement of blood alcohol concentration (BAC) after administration of a single high dose of alcohol (3.0 mg/kg, *i.p.)* to alcohol-naive group-housed adolescent mice (PND 36–42) did not provide any evidence for an altered alcohol metabolism in the transgenic mouse line (*p* = 0.784; Fig. 3f). In addition, no difference in the sedating effects of ethanol was found between adolescent wt and transgenic mice using the loss of righting reflex (LORR) paradigm (Fig. 3g). Finally, we excluded that, owing to the transient social isolation, single-housing (SH) was a confounding factor per se by comparing EtOH-induced currents between GCs from adult alcohol-naive mice either group-housed all their life or subjected to 2 weeks of SH during their adolescence (Supplementary Fig. 1b).

**Fig. 4:**
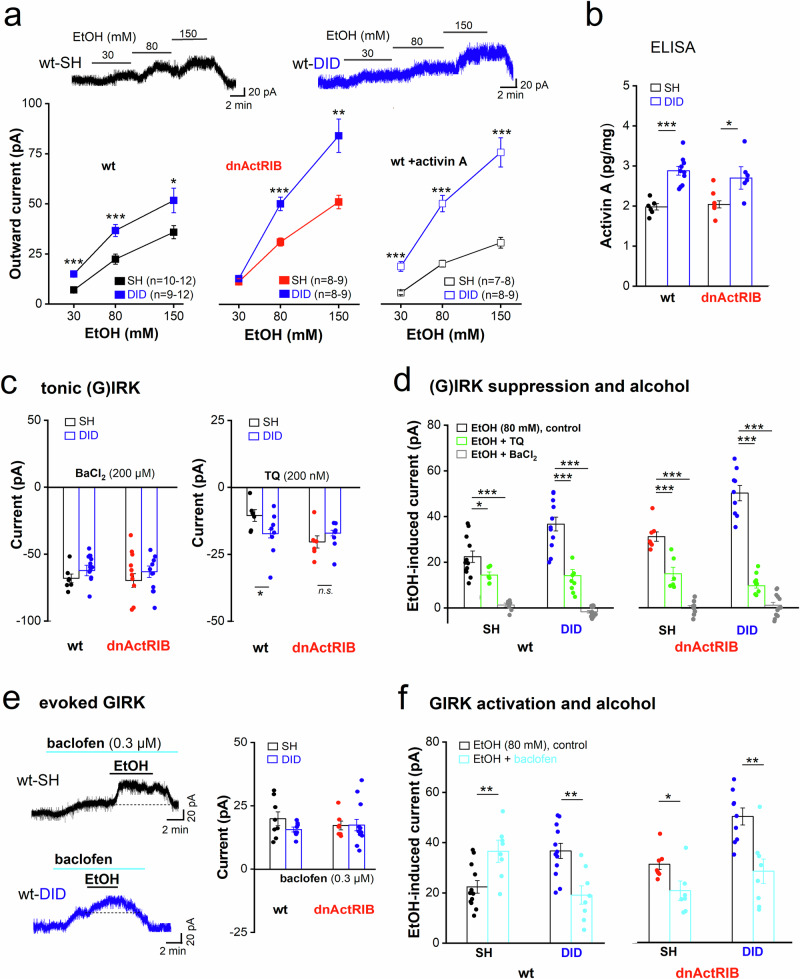
Impact of adolescent drinking on adult alcohol response involves activin A and GIRK channel functionality. (**a**) Dose-response curves demonstrate augmenting effect of DID on acute EtOH application in GCs from adult mice of either genotype. Incubating wt-DID slices with activin A (25 ng/ml for 3–6 h) further enlarged EtOH response. Traces above depict outward current responses to increasing EtOH concentrations in GCs from adult wt mice with adolescent SH (black trace) or DID experience (blue trace). **(b)** ELISA measurements reveal higher activin A protein levels in hippocampi from DID-exposed adult wt and dnActRIB mice adults compared to alcohol-naive littermates. **(c)** Histograms summarize tonic Ba^2+^- and TQ-sensitive currents in GCs from the different groups. (**d**) Histograms summarize TQ-sensitive fraction of total EtOH-evoked current in GCs from the groups as indicated by like-colored letters above columns. Ba^2+^ fully suppressed EtOH response in all groups. **(e)** Traces depict GIRK current activation by additive application of baclofen (0.3 µM) and EtOH (80 mM EtOH) in GCs from wt control (SH, upper trace) and wt DID mouse (lower trace). Histogram on right shows effect of baclofen alone. (**f**) Histograms compare GIRK current response to EtOH alone or in the presence of pre-applied baclofen. Statistical comparisons were performed using an unpaired, two-tailed student’s t-test at α = 0.05 (**b, c, e, f**) or a one or two-way ANOVA followed by Tukey’s post-hoc test (**a,** **d**). * *p* < 0.05; ** *p* < 0.01; *** *p* < 0.001.

In GCs from adult wt mice with a history of heavy adolescent drinking (wt-DID), EtOH produced significantly larger outward currents than in cells from age-matched alcohol-naive mice (Fig. 4a, left**)**: 30 mM, 14.9 ± 1.5 pA, *n* = 12, *p* = 3.90e^−4^; 80 mM, 36.7 ± 3.0 pA, *n* = 12, *p* = 0.001; 150 mM, 51.7 ± 6.2 pA, *n* = 9, *p* = 0.021; compared to the respective wt-SHs). The enhanced alcohol responsiveness after DID was even more pronounced, at least for EtOH ≥ 80 mM, when we compared EtOH currents between GCs from adult transgenic mice with and without preceding DID experience (Fig. 4a, middle**:** dnActRIB-DID, 80 mM, 50.2 ± 3.4 pA, *n* = 9, *p* = 1.53e^−5^; 150 mM: 84.2 ± 8.3 pA, *n* = 8, *p* = 0.002; compared to the respective dnActRIB-SHs). The stronger EtOH response after DID was accompanied by a strong rise in activin A, lending further credence to the idea that the factor controls the impact of alcohol in the brain (adult wt-DID, 2.9 ± 0.11 pg/mg, *n* = 10, *p* = 3.26e^−5^; adult dnActRIB-DID, 2.7 ± 0.3 pg/mg, *n* = 6, *p* = 0.017; compared to their respective alcohol-naive littermates; Fig. 4b). Interestingly, preincubation of wt-DID adult slices with activin A (25 ng/ml for 3–6 h) further enhanced the acute effect of ethanol (Fig. 4a, right: wt-DID in activin, 80 mM, 50.1 ± 4.1 pA, *n* = 8, *p* = 0.014 vs adult wt-DID; *p* = 2.09e^−5^ vs wt-SH in activin). As in granule cells from untreated mice (Fig. 2a, b), ramp-evoked EtOH-sensitive current reversed polarity around E_K_ in all groups with adolescent SH and/or DID experience (*data not shown)*.

We next examined whether DID would alter total IRK current and/or the fraction of GIRK current therein. First, we demonstrated that Ba^2+^- and TQ-sensitive currents did not differ between adult GCs from SH- and GH-mice of both genotypes, underscoring the validity of SH mice as proper controls for the DID group (Supplementary Fig. 1c). The low Ba^2+^-sensitive standing current was about equal between GCs from adult DID and SH mice of either genotype (Fig. 4c). With respect to tonic GIRK current, however, DID enhanced the TQ-sensitive current only in wt GCs, but not in mutant GCs, possibly reflecting a ceiling effect, given that the TQ-sensitive current was already larger in GCs of mutant SH mice (wt-DID TQ, −17.3 ± 2.9 pA, *n* = 8; wt-SH TQ, −10.5 ± 2.2 pA, *n* = 6, *p* = 0.024; dnActRIB-SH, TQ alone, −20.3 ± 2.9 pA, *n* = 7; dnActRIB-DID, TQ alone −18.0 ± 1.7 pA, *n* = 8; *p* = 0.416; Fig. 4c). In all groups, EtOH (80 mM)-evoked currents were completely blocked by low Ba^2+^ (Fig. 4d). Application of TQ revealed that adolescent DID enhanced the TQ-sensitive current, especially in GCs from transgenic DID mice, in which it accounted for approximately 80% of the EtOH response (dnActRIB-DID: EtOH 80 mM in TQ, 9.6 ± 1.0 pA, *n* = 6; EtOH alone 50.2 ± 3.4 pA, *n* = 7; *p* = 9.47e^−5^, Fig. 4d).

Given the large degree of variability in TQ sensitivity between different experimental groups, it remains to be determined how activin A and/or DID alter the assembly of functional GIRK channels. As a first step to address this issue, we examined the transcriptional regulation of *KCNJ3* and *KCNJ6*, which encode GIRK1 and GIRK2, respectively. However, qPCR measurements did not reveal significant differences in expression levels between SH and DID groups in either genotype (Supplementary Fig. 3). It is therefore unlikely that DID and/or activin affected transcription of the channel genes.

Notably, DID left the G_i/o_ pathway of GIRK activation apparently unchanged, as GIRK currents evoked by the GABA_B_R agonist baclofen (0.3 µM) did not significantly differ between GCs from adult SH and DID mice of either genotype (Fig. 4e, right). Unexpectedly, however, pre-administration of baclofen did affect the size of the subsequent EtOH current, which was superimposed on the steady-state baclofen-induced current. In wt controls (SH), the relatively small EtOH response was augmented in the presence of baclofen, whereas the substantially larger EtOH current after DID was significantly diminished by pre-applied baclofen (wt-SH, EtOH in baclofen: 36.5 ± 4.4 pA, *n* = 12; EtOH alone, 22.4 ± 2.5 pA, *n* = 12, *p* = 0.008, wt-DID: EtOH in baclofen: 19.1 ± 3.6 pA, *n* = 8; EtOH alone: 36.7 ± 3.0 pA, *n* = 12, *p* = 0.009 Fig. 4e, f). In GCs from transgenic SH and DID mice, which exhibited already larger EtOH responses than the respective wt GCs, baclofen reduced EtOH currents in both groups (dnActRIB-SH: EtOH in baclofen, 20.6 ± 3.9 pA, *n* = 6; EtOH alone 31.1 ± 2.0 pA, *n* = 8, *p* = 0.024; dnActRIB-DID: EtOH in baclofen, 28.4 ± 4.9 pA, *n* = 8; EtOH alone 50.2 ± 3.4 pA, *n* = 9, *p* = 0.002) (Fig. 4f). In a similar vein, heavy adolescent drinking did not alter the GIRK response to ML 297 per se (Supplementary Fig. 4a). However, just as for baclofen, pre-activation of a fraction of GIRK channels by ML 297 reduced the sensitivity of GCs to subsequent EtOH in the respective groups (wt DID mice, transgenic mutant SH and DID mice, Supplementary Fig. 4b).

### Adolescent drinking strengthens the inhibitory effect of alcohol on cell firing in adulthood

**Fig. 5:**
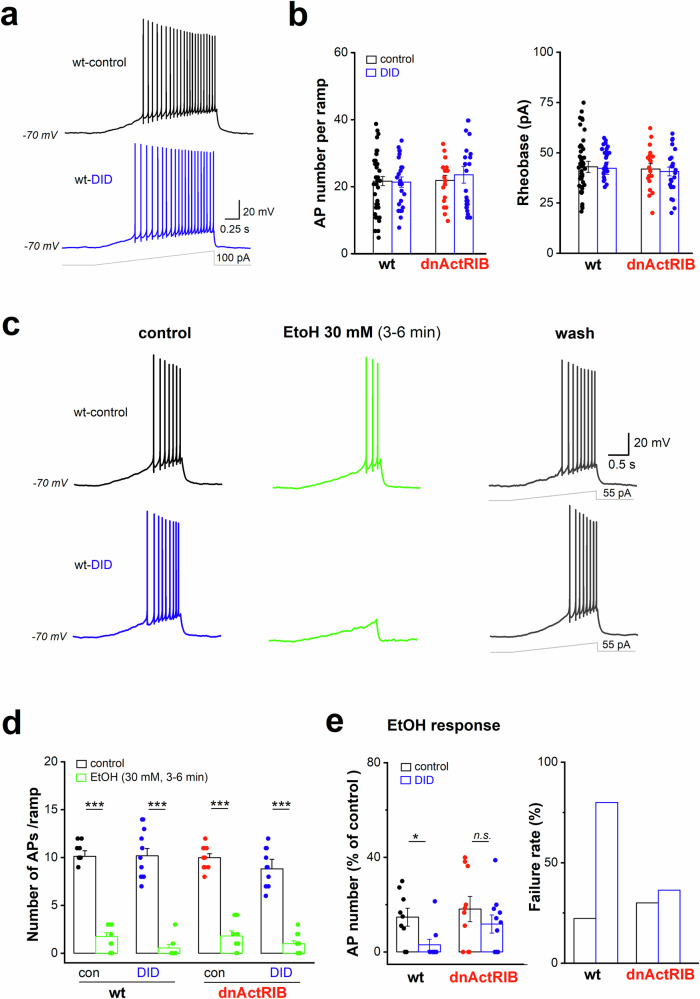
Adolescent binge-like drinking strengthens inhibitory effect of EtOH on GC firing in adulthood. Whole-cell current-clamp recordings were performed from GCs in slices from adult wt and dnActRIB mice with or without adolescent drinking experience (DID). Cells were held at −70 mV by injection of depolarizing current, and were pharmacologically isolated from GABAergic and glutamatergic inputs by PTX (100 µM) and KA (2 mM), respectively. **(a-b)** Action potential (AP) firing was examined using a depolarizing current ramp (0 to 100 pA within 2 s), as illustrated in **a** and summarized in **b**. (**c-e**) DID enhanced EtOH-mediated inhibition of firing in adulthood. Recordings in **c** are from wt GCs, showing reversible suppression of firing upon acute EtOH exposure (30 mM). The depolarizing ramp current was individually adjusted before alcohol application to induce 6–12 APs, with membrane potential initially held at −70 mV. Histograms in **d** summarize the massive reduction of AP discharge by EtOH in all groups. In GCs from adult wt mice with preceding DID, EtOH-induced firing suppression was more powerful than in GCs from alcohol-naive mice, as shown in (**e**), where the response of individual cell was normalized to percentage of control value (left columns), or expressed as failure to evoke APs during maximum EtOH response (right columns). Statistical comparisons were performed using paired- or unpaired, two-tailed student’s t-test at α = 0.05. *n.s*. not significant; * *p* < 0.05; *** *p* < 0.001.

In whole-cell current-clamp recordings with blockers of fast excitatory and inhibitory transmission, GCs from all four groups displayed comparable passive electrophysiological properties, including resting membrane potential (RMP), membrane input resistance and capacitance (Table 1). GCs did also not differ with regard to core features of cellular excitability such as firing pattern and rheobase (Fig. 5a, b). In GCs from adult control mice, EtOH (30 mM) produced a strong suppression of ramp-evoked firing (*n* = 9, from 10.6 ± 0.3 APs to 1.6 ± 0.4 APs per ramp, *p* = 9.3e^−8^, paired t-test; Fig. 5d). As expected from the unchanged GIRK current response to 30 mM EtOH (Fig. 4a), a preceding DID history did not affect suppression of AP firing by the same concentration of EtOH in GCs of adult mutant mice (Fig. 5d, e). However, when re-exposing GCs from adult wt DID mice to EtOH (30 mM), we observed a significantly stronger suppression of evoked firing compared to their alcohol-naive counterparts (Fig. 5c, d), consistent with the enhanced GIRK current response to 30 mM EtOH after DID (Fig. 4a). In the majority of GCs (8 out of 10) of wt DID mice, depolarizing ramps failed to elicit even a single AP in the presence of 30 mM EtOH (failure rate of 80%; Fig. 5c–e). In contrast, 7 out of 9 wt-control cells still produced few APs in the same experimental setting (wt-control, failure rate 22%; Fig. 5c–e). Expressed as relative decline, 30 mM EtOH reduced the number of ramp-evoked APs to 14.7 ± 3.8% in wt-control slices (*n* = 9), whereas in wt-DID slices, AP firing was almost completely silenced to 3.0 ± 2.3% (*n* = 10), which was significantly lower (*p* = 0.016; Fig. 5e). The concomitant hyperpolarization of GCs from wt DID mice (−4.8 ± 0.5 mV) was comparable with that of wt controls (−5.0 ± 0.6 mV, *p* = 0.928). These data suggest that, with a history of heavy adolescent drinking, a single drinking bout later in life will dampen neuronal excitability more effectively than in alcohol-naive adults.

**Table 1:** Passive membrane properties of dorsal dentate gyrus granule cells from adult wt and dnActRIB mice.

DG GCs	wt control (n = 42)	wt DID (n = 24)	dnActRIB control (n = 20)	dnActRIB DID (n = 22)
RMP (mV)	−85.77 ± 0.54	−85.24 ± 0.78	−86.61 ± 0.88	−86.47 ± 0.68
Input resistance (MΩ)	299.27 ± 9.68	313.13 ± 12.86	293.00 ± 10.00	297.95 ± 12.86
Capacitance (pF)	93.00 ± 2.26	91.26 ± 3.07	100.10 ± 3.36	100.50 ± 3.95

Data were collected few minutes after whole-cell formation in voltage-clamp mode (−70 mV).

## Discussion

In the present study, we focused on GIRK channels as actuators of the neuronal response of hippocampal granule cells (GCs) to alcohol consumption. GCs are recognized for their heightened sensitivity to low concentrations of alcohol, which has been attributed so far to the expression of δ subunit-containing GABA_A_Rs [28, 29]. Notwithstanding the established role of this GABA_A_ receptor subtype in mediating the effects of low EtOH, our study suggests that the interaction between activin signaling and GIRK current makes also a substantial contribution to render GCs particularly responsive to EtOH in the low millimolar range. Regarding the minimum effective concentration of acute EtOH, we noted indeed a remarkably low threshold in GCs from adolescent alcohol-naive mice, with considerable GIRK current activation at 15 mM EtOH, corresponding to 0.07% blood alcohol concentration (BAC). To illustrate, a 16-year-old male with 60 kg body weight would reach such a BAC level by drinking 1 l beer (5%) within one hour. In contrast, GIRK current in GCs from adult mice proved insensitive to 15 mM EtOH, which might explain why GIRK channels are missing so far on the list of molecular targets of low-dose alcohol [46].

Owing to its effects on general membrane properties such as fluidity and its limited potential (as a two-carbon molecule) to interact with other biomolecules, EtOH has a reputation as a nonspecific drug. However, to quote from the above-mentioned review by Abrahao et al. [7], “the earlier ideas about ubiquitous effects due to changes in membrane fluidity are not helpful in understanding how ethanol alters neuronal function”. In order to be classified as a direct molecular target of ethanol, the putative target protein has to fulfil a number of pharmacological, biochemical, and structural criteria, which are outlined in the review by Abrahao et al. [7]. Since GIRK channels are among the direct molecular targets, we would argue that we are dealing with a specific effect of ethanol on neuronal excitability.

During adolescence, the hippocampus is a unique target of alcohol, as adolescents appear to be more vulnerable than adults to the detrimental effects of alcohol on learning and memory. Encoding of new explicit memories shows a particularly strong impairment, whereas recall of established memory content or working memory functions are much less affected [47]. Thus, alcohol disrupts primarily long-term synaptic plasticity, the presumed mechanism underlying the formation of new memory traces. At most excitatory CNS synapses, induction of lasting changes in transmission efficacy requires activation of NMDA receptors. Of note, NMDA receptors are among the molecular targets inhibited by alcohol [7]. Nevertheless, the concomitant enhancement of GIRK currents by alcohol should also make a sizeable contribution to hampered learning, the more so as GIRK channels reside in dendritic shafts and spines near excitatory synapses [48, 49]. With this strategic location, they are ideally poised to counteract the depolarization-induced activation of NMDA receptors [49–52].

The heightened susceptibility of GCs from young mice to low-dose alcohol coincides with the augmenting effect of activin A on ethanol responses at this age. The remarkable constellation implicates the setting, in which alcohol is consumed, in how strong the drug will act on an individual. This idea stems from our previous observation that behavioral stimulation, e.g. exploration of a novel and enrichment environment, engenders a pronounced up-regulation of hippocampal activin A [38, 39]. Thus, an appealing, spirit-lifting experience with its accompanying surge in endogenous activin A might potentiate the characteristic behavioral disinhibition associated with BAC levels between 0.03 and 0.08% [46].

Previous work from our and other labs on activin signaling in the hippocampus under physiological conditions showed that the levels of *Inhba* mRNA, which gives rise to the β_A_ subunit of homodimeric Activin A (β_A_β_A_), or of Activin A protein are strongly regulated by neural activity, be it induced by behavioral stimulation such as a 12 h exploration of a new and enriched environment, or by direct electrical stimulation of excitatory fiber tracts within the hippocampal formation [38, 39, 53, 54]. Whereas in the absence of such stimulation, the level of endogenous activin A is rather low, it exhibits a remarkable stimulation-induced surge, which peaks within few hours and then declines again. When we incubated slices with recombinant activin A for 3–6 h, we thus mimic the physiological situation of transiently elevated activin signaling. Thus, our comparison between EtOH-related gating in the absence and presence of recombinant activin A should inform about physiological changes in how EtOH controls GIRK channel activity in relation to the current state of activin signaling.

In the brain, activin signaling is predominantly mediated by ActRIB-containing receptor complexes and may involve both canonical and non-canonical pathways [31]. In a previous study, we linked the increase in granule cell firing after overnight environmental enrichment (EE) to a rise in endogenous activin A, which in turn reduces a standing GIRK current to make granule cells more excitable [36]. In that study, the increase in GC firing observed after EE exploration was faithfully reproduced by recombinant activin A in hippocampal slices from control animals independent of whether the factor was pre-incubated for hours or acutely applied within minutes. The latter regimen would not be compatible with a signaling pathway involving gene transcription. Moreover, the excitatory effect of activin A was preserved when the factor was pre-incubated with a blocker of SMAD signaling (SB 431542) but was abrogated by a selective ERK1/2 MAPK inhibitor (PD 98058) and a selective PKA inhibitor (KT 5720). These data suggest that activin A can reduce GIRK current at short notice through non-canonical signaling.

The dominant-negative activin receptor IB mouse model disrupts activin signaling without interfering with signaling by other members of the TGFβ family. We used this model here, because it has become a valuable tool to elucidate the many facets of activin signaling in the normal and diseased brain, ever since it was generated by our collaborators [37]. As an important asset of this mouse model, the transgene is expressed under the control of the CaMKIIα promoter, which does not become activated before the second postnatal week; thus, the mouse does not exhibit a developmental phenotype. Furthermore, expression of the transgene is largely confined to principal (excitatory) neurons of the forebrain. The functionality of the transgene has already been demonstrated for canonical (Smad-dependent) activin signaling [37] as well as for non-canonical effects of the transgene gene that may be mediated by MAPK or PKA/C [30, 36]. Importantly, previous work has shown that the transgene does not alter the level and release of endogenous activin A [36, 53]. Specifically, we included the dnActRIB mouse in this study, because we had previously found that activin signaling (i) alters behavioral effects of alcohol [30] and (ii) inhibits GIRK current, which is an established molecular target of EtOH [36].

Since activin A excites adult GCs through inhibition of GIRK current [36], we propose that activin decreases the EtOH response in the adult preparation by reducing the availability of GIRK channels. In adulthood, the standing, TQ-sensitive GIRK current was indeed down-regulated by activin A in wt hippocampus and enhanced in dnActRIB hippocampus. Moreover, in the adolescent preparation, where activin A boosted the EtOH response, we measured a significantly higher tonic GIRK current after pre-incubation with activin A. Mechanistically, activin A might regulate GIRK channel availability in several ways, including, among others, (i) transcription of *KCNJ6*, which encodes the prevailing GIRK2 subunit, (ii) channel surface expression, and (iii) silencing of membrane-inserted channels.

A clue comes from the effect of the GIRK channel activator ML 297 on the EtOH response in adolescent GCs from dnActRIB mice. If measured in the presence of ML 297, the EtOH-evoked GIRK current, which used to be smaller compared to that in wt cells (see above), was now massively enhanced. This finding suggests that the absence of activin signaling renders GIRK channels in GCs of adolescent mutant mice insensitive to EtOH. Only after being “unmuted” by ML 297, they are able to react to EtOH.

The effect of activin signaling on GIRK current should be particularly powerful, as our earlier work strongly suggests that it occurs most likely downstream of the many modulator systems that converge onto GIRK channels [36]. These channels are widely recognized as a prominent molecular target of EtOH. In a nutshell, we posit here that the way activin A and EtOH regulate GIRK current can be strikingly divergent: In the adolescent brain and, most importantly, in the adult brain after adolescent binge drinking, activin and EtOH work together as synergistic activators of GIRK current. In sharp contrast, in the adult brain of alcohol-naïve mice, activin A and EtOH assume the role of opponents, reducing and augmenting, respectively, GIRK current. Thus, examination of the interplay of the threesome consisting of activin A, EtOH and GIRK channel and their lasting alterations after adolescent binge drinking offers new insights into a (patho)-physiologically relevant mechanism.

Obviously, GIRK channels are not the only target of activin signaling. In fact, we are tempted to consider activin signaling as a kind of “master molecule” that optimize, through its joint action on NMDA receptors, GABA_A_ receptors and GIRK channels the performance of hippocampal networks. Just like GIRK channels, NMDA receptors and GABA_A_ receptors have been identified as molecular targets of EtOH [7]. Thus, our GIRK-EtOH-activin study does not give a full account of how activin signaling modulates the alcohol-besieged brain. However, we believe that the interactions between EtOH, GIRK channels and activin A are sufficiently intriguing to be singled out. Although we cannot exclude that activin signaling acts also on cholinergic, noradrenergic or dopaminergic systems, we would argue that such effects should not interfere with the interpretation of this study as far as it relates to GIRK channels, since activin A most likely targets channel properties in a direct fashion, thereby rendering possible upstream actions less relevant.

In a similar vein, we are aware that, by focusing on the hippocampus, our study does not offer a comprehensive understanding of how adolescent binge drinking imprints lasting alterations on brain functions. To achieve such a global view, further studies would be required in other brain regions, with particular emphasis on those involved in the control of ethanol consumption, such as the prefrontal and insular cortex and other structures of the reward system [55, 56].

Using the drinking-in-the-dark (DID) paradigm, we found that a history of heavy alcohol consumption during adolescence leaves a stamp on how the adult brain deals with acute EtOH re-exposure. Despite a drug-free interval of 45–105 days, GCs from DID mice showed a significantly stronger GIRK current response to EtOH than cells from the respective control mice. For high EtOH doses, long-lasting sensitization by earlier DID was even more pronounced in mutant GCs than in wt GCs, although dnActRIB mice consumed less EtOH during DID than their wt counterparts. Interestingly, the way in which alcohol abuse affects GIRK channel activity shows a strong dependence on the type of aberrant drinking behavior. Whereas our binge drinking paradigm that was restricted to a 2-week period in adolescence followed by a long drug-free interval, sensitized GIRK channels to renewed EtOH ingestion in adulthood, chronic alcohol consumption entails the opposite effect, namely a blunting of GIRK function [57].

Low Ba^2+^, an unselective blocker of inwardly rectifying K^+^ channels, fully and reliably suppressed EtOH-evoked GIRK channel activity in all sets of experiments. In contrast, the GIRK channel blocker TQ inhibited only a fraction of the GIRK current response, albeit with remarkable variability, ranging from virtually no inhibition, such as observed in GCs from adolescent dnActRIB mice, to almost complete inhibition, such as seen in GCs from adult dnActRIB mice with preceding DID experience. Since EtOH does not affect IRK1 (Kir2) [15, 58], it is unlikely that the TQ-resistant component of the EtOH-associated outward current is carried by Kir2 channels. Rather, we have to consider the possibility that the GIRK current evoked by EtOH and the GIRK current blocked by TQ are mediated by GIRK channel populations with a variable degree of overlap. In fact, TQ binding to GIRK1 channels [41], which are unable to form homomers, is supposed to inhibit GIRK1/2 heteromers, presumably the most prevalent GIRK configuration in GCs, but it should not suppress currents arising from GIRK2 homomers or GIRK2/3 heteromers. By contrast, the binding pocket for EtOH is located in the GIRK2 subunit [19], suggesting that EtOH is poised to activate all GIRK2-containing channel combinations. Thus, the larger the TQ-sensitive fraction of the overall GIRK current response is, the more it should be carried by GIRK1/2 heteromers, and perhaps also GIRK1/3 heteromers.

GIRK channels are not the only target through which alcohol can affect intrinsic excitability. Other K^+^ channels involved in the control of cellular firing, whose activity is also subject to alcohol abuse, are K_V_7 (KCNQ) and K_Ca_2 (SK) channels [59]. Moreover, HCN channels and L-type Ca^2+^ channels are modulated by alcohol, too [7]. We have not examined these channel types with respect to a lasting impact of adolescent DID. Nevertheless, the fact that adult GCs from DID and control mice did not differ in basic electrophysiological properties nor in their firing behavior would argue against gross abnormalities in any of the above channels. Thus, it seems reasonable to explain the enhanced inhibition of firing by acute EtOH in adult mice after earlier binge drinking by the DID-associated amplification of GIRK current responsiveness.

Our finding that administration of baclofen reversed the DID-induced hyper-responsiveness to alcohol later in life should be of translational interest, given that the drug is in clinical use to treat AUD [27]. So far, the therapeutic effects of baclofen in alcohol patients and rodent models thereof have been attributed to the hyperpolarization of mesolimbic dopamine neurons in the ventral tegmental area (VTA) as well as to the normalization of tonic inhibition in the amygdala through presynaptic GABA_B_ receptors [60]. Here, we show that baclofen is also capable of counteracting the enhanced sensitivity of GIRK channels to ethanol after binge drinking in early ages. This finding introduces not only a putative new mechanism of therapeutic action, but, with the hippocampus, also a new site of action, with direct implications for AUD-associated cognitive deficits and affective disorders. It is noteworthy that, apart from France, the use of baclofen in AUD is “off-label” and remains controversial due to issues regarding dosing, efficacy and unwanted side effects [26]. The fact that, in our hands, the direct GIRK channel activator ML 297 was as effective as baclofen in counteracting the exaggerated ethanol response holds promise for the development of more precise drugs against AUD.

## Supplementary information

supplemental material

## Data Availability

Data of this study are available from the corresponding author upon reasonable request.
