# Supplementary material for: Heavy adolescent drinking makes the adult brain more vulnerable to ethanol by permanently altering the age-dependent interplay between alcohol, GIRK channels and activin
Source: Mol Psychiatry. 2025 Sep 3;31(2):1027–40. doi: 10.1038/s41380-025-03210-x (PMC12815657; doi:10.1038/s41380-025-03210-x)
Supplement: Supplementary file 1 — supplemental material [file 41380_2025_3210_MOESM1_ESM.docx]

**Heavy adolescent drinking makes the adult brain more vulnerable to ethanol by permanently altering the age-dependent interplay**

**between alcohol, GIRK channels and activin.**

Sophia Stürzenberger^#1^, Nicolas Bülow^#1^, Liubov S. Kalinichenko^2^, Rebecca Licha^2^, Volker Eulenburg^3^, Marc Dahlmanns^1^, Christian P. Müller^2,4^, Fang Zheng*^1^, Christian Alzheimer*^1^

Supplementary information

**
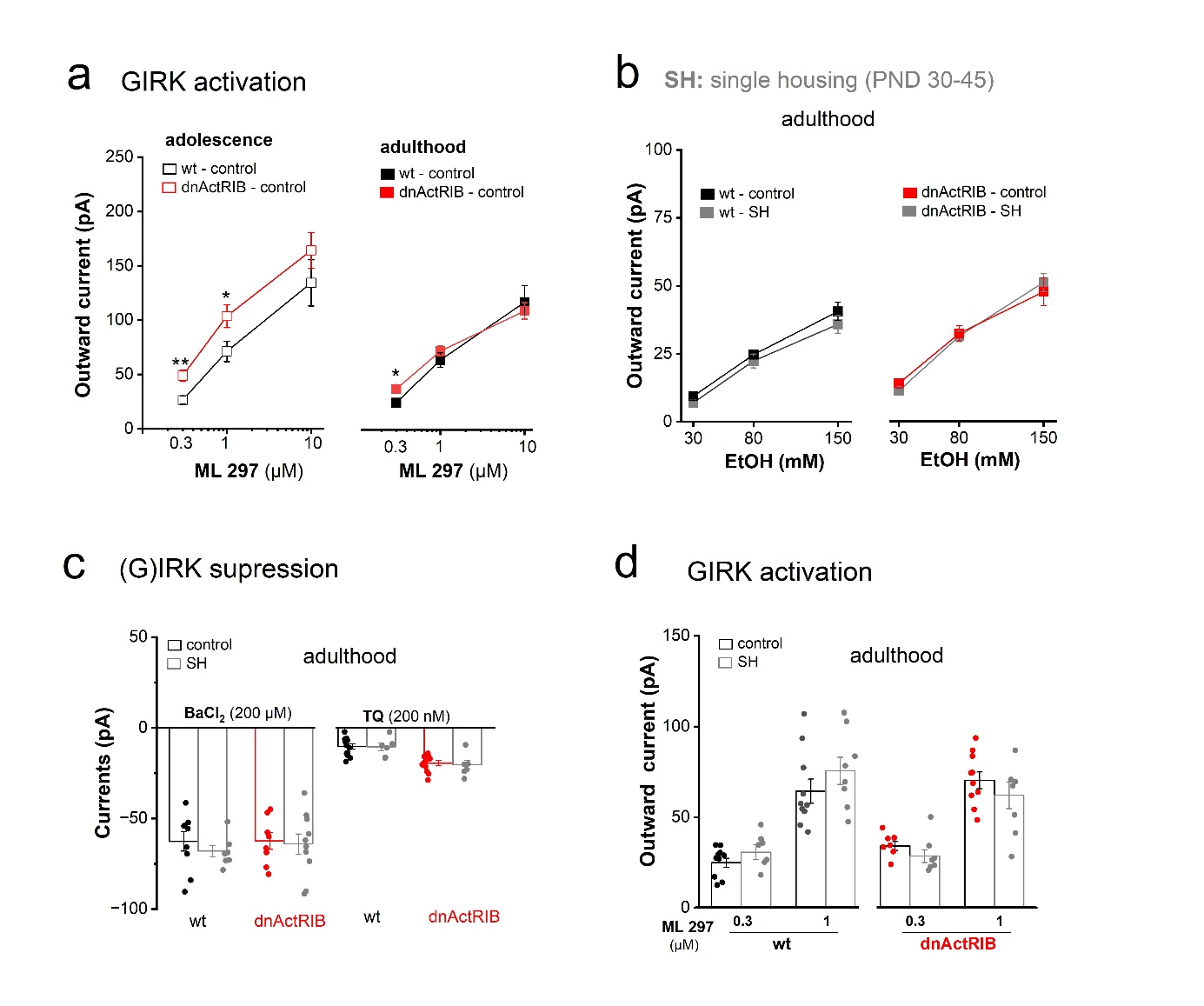
**

**Supplementary Fig. 1** (**a**) Dose response curves for outward current response to ML 297 in voltage-clamped GCs (V_h_ -70 mV) in slices from adolescent and adult mice of either genotype. Adolescents: wt n = 7-9, dnActRIB n = 7-10. Adults: wt n = 7-10, dnActRIB n = 7-11. (**b-d**) Comparison of GIRK responses to ethanol, of Ba^2+^- and TQ-sensitive current, and of ML 297-induced current between GCs from adult GH mice (control) and adult mice with adolescent SH experience (without alcohol exposure) reveals no significant effect of SH in either genotype (Adults: wt-control n = 9-10, dnActRIB-control n = 7-8; wt-SH n = 10-12, dnActRIB-SH n = 8-9). Statistical comparisons were performed using an unpaired, two-tailed student’s t-test at α = 0.05 (c, d) or a two-way ANOVA followed by Tukey’s post-hoc test**. * p < 0.05.**

**
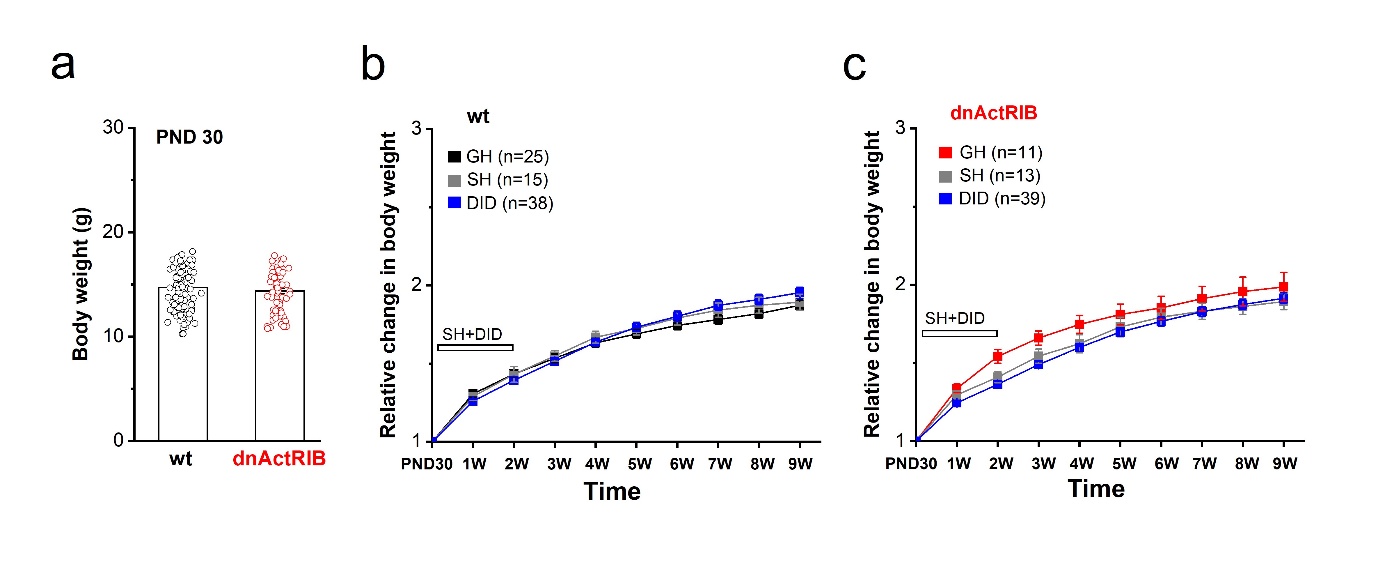
**

**Supplementary Fig. 2 (a)** Comparable body weight of wt and dnActRIB mice at PND 30. (**b-c**) Relative increase in body weight after onset of DID in the different groups was normalized to weight at PND 30. Body weight was measured every week (W).

**
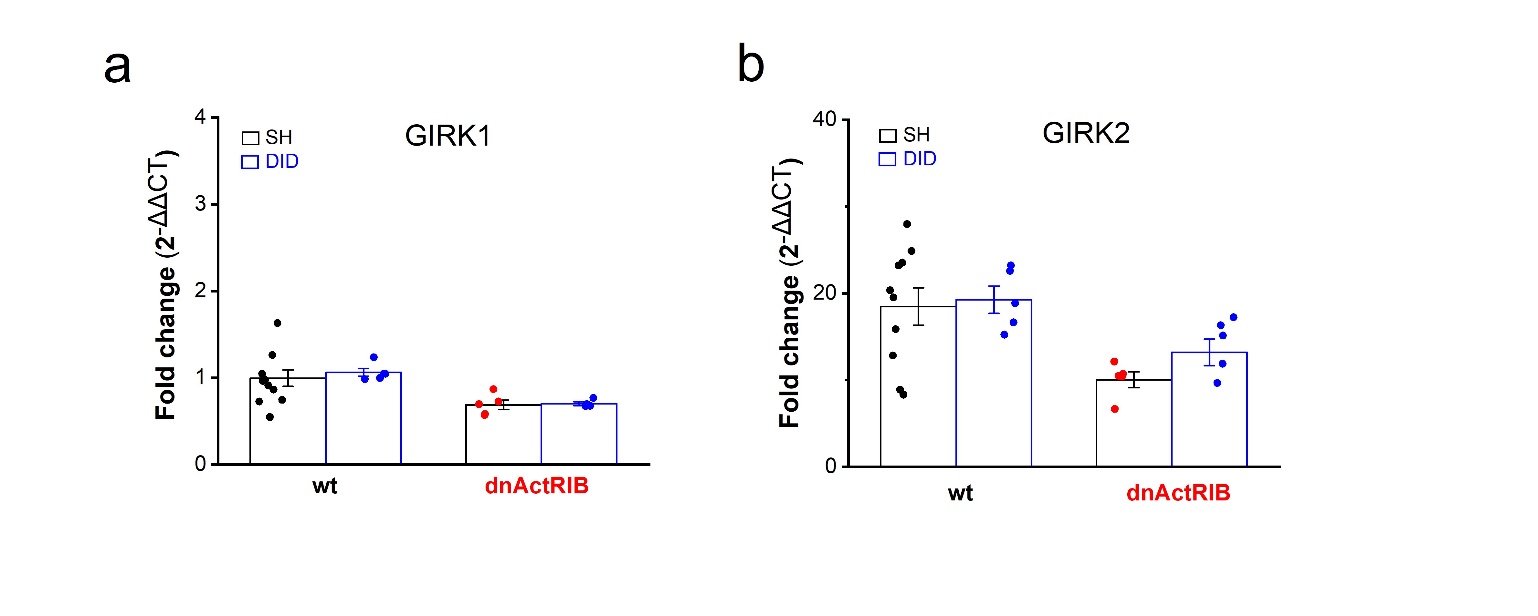
**

**Supplementary Fig. 3 RT-qPCR analysis of GIRK1 subunit expression (a) and GIRK2 subunit expression (b) in dorsal dentate gyrus from adult wt and dnActRIB mice with and without adolescent DID.**

**
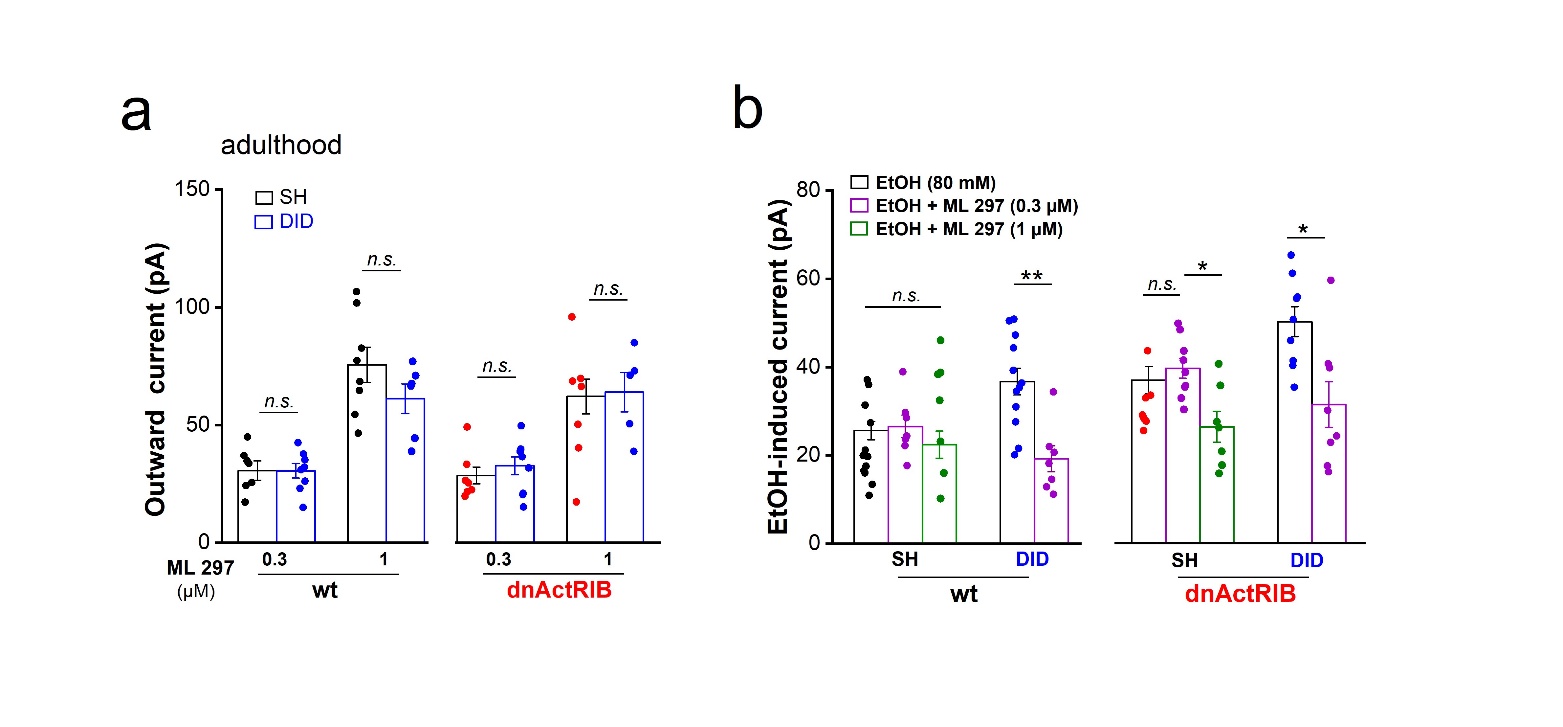
**

**Supplementary Fig. 4 (a)** GIRK channel sensitivity to ML 297 was not altered by DID in GCs from adult wt and dnActRIB mice. (**b**) The DID-enhanced GIRK response to EtOH in GCs from adult wt and dnActRIB mice was reversed by pre-applied ML 297(0.3 µM). Statistical comparisons were performed using sing an unpaired, two-tailed student’s t-test at α = 0.05 or a one-way ANOVA followed by Tukey’s post-hoc test. n.s., not significant, * p < 0.05; ** p < 0.01.
